# Multifunctional Liposomes: Smart Nanomaterials for Enhanced Photodynamic Therapy

**DOI:** 10.3390/biomimetics10100689

**Published:** 2025-10-13

**Authors:** Ji-Won Yu, Do Gyun Kim, Gi Doo Cha

**Affiliations:** Department of Systems Biotechnology, Chung-Ang University, Anseong 17546, Republic of Korea; yujiwon621@cau.ac.kr (J.-W.Y.); kdg4376@cau.ac.kr (D.G.K.)

**Keywords:** liposome, cancer, photodynamic therapy, theranostics, immunotherapy, nanomedicine

## Abstract

Cancer remains one of the leading causes of mortality worldwide and continues to pose significant therapeutic challenges despite decades of research. Conventional treatments such as chemotherapy and radiotherapy often lack selectivity, damaging both malignant and healthy tissues and resulting in severe side effects. Photodynamic therapy (PDT) has emerged as a promising non-invasive alternative that selectively eradicates cancer cells or pathogens using a photosensitizer (PS), light, and oxygen. PDT induces necrosis or apoptosis in cancer cells by locally generating cytotoxic reactive oxygen species through targeted laser irradiation. However, its clinical efficacy is limited by factors such as tumor hypoxia, poor PS delivery efficiency, and light attenuation within biological tissues. Recent advances in liposomal nanoplatforms have shown considerable potential in overcoming these barriers. Liposomes can co-deliver PS, therapeutic agents, and oxygen, thereby enhancing PDT outcomes. This review outlines the fundamental principles of PDT and the physicochemical properties of liposomes. It then explores two major strategies for improving PDT efficacy using liposomes: PS-drug co-delivery and oxygen delivery to mitigate tumor hypoxia for synergistic therapeutic effects. Finally, current limitations and future perspectives of liposome-based nanomedicine in photodynamic cancer therapy are discussed. Overall, this review provides a foundation for advancing liposome-based strategies toward clinical implementation in photodynamic cancer treatment.

## 1. Introduction

Cancer remains one of the leading causes of mortality worldwide, with over 18 million new cases diagnosed annually and approximately 9.5 million cancer-related deaths reported in 2018 [1,2]. Surgical resection, while effective in removing large tumor masses, is limited by the potential for recurrence due to residual malignant cells [3,4]. Other conventional therapies, such as chemotherapy and radiotherapy, are capable of destroying cancer cells but often cause significant collateral damage to healthy tissues [4,5].

**Figure 1:**
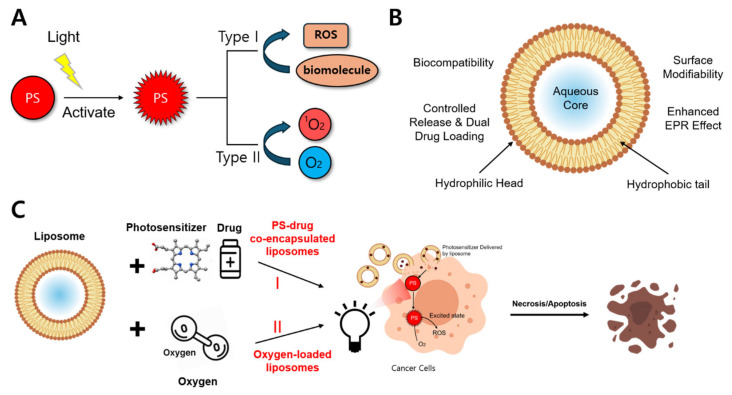
Schematic overview of liposome-assisted photodynamic therapy (PDT). (**A**) NIR-activated PS generates cytotoxic species via Type I or Type II mechanisms. (**B**) Liposomes encapsulate various therapeutic agents and enhance targeting via structural and surface properties. (**C**) Liposomes co-deliver PS, drugs, and oxygen to tumor cells, enhancing PDT-induced cell death.

To overcome these limitations, photodynamic therapy (PDT) has emerged as a promising non-invasive alternative for cancer treatment [6,7,8]. PDT involves the use of a non-toxic photosensitizer (PS) and harmless near-infrared (NIR) light. Upon NIR irradiation, the PS becomes excited and transfers energy either to surrounding biomolecules-producing reactive oxygen species (ROS) such as superoxide or hydroxyl radicals (Type I)-or to molecular oxygen, generating singlet oxygen (1O_2_, Type II), which serves as the predominant cytotoxic agent [9,10,11]. These ROS indiscriminately damage cellular components, including proteins, lipids, and nucleic acids, leading to cell death and vascular disruption, ultimately destroying tumor tissues (Figure 1A) [10,11,12].

Compared to conventional therapies, PDT offers localized treatment with reduced systemic toxicity due to its spatially controlled activation by light irradiation [13,14]. However, its therapeutic efficacy is constrained by several factors, including tumor hypoxia [15,16], inefficient PS delivery [15,16], and limited light penetration in biological tissues [10,17]. Moreover, ROS generated during PDT exhibit a short half-life and a restricted diffusion radius of approximately 10–55 nm, confining their cytotoxic effects to regions directly exposed to light [9,10].

To overcome these challenges, various material-based strategies have been explored, including supramolecular PS assemblies, nanoparticles, and liposomes [18,19,20]. Among these, liposomes-composed of phospholipid bilayers have garnered significant attention due to their excellent biocompatibility, structural stability, and biodegradability [21]. Their amphiphilic architecture enables the encapsulation of hydrophilic agents within the aqueous core and the incorporation of hydrophobic compounds into the lipid bilayer, enabling efficient co-delivery of therapeutic molecules and facilitating dual drug loading (Figure 1B) [22,23,24]. Leveraging these advantages, liposomes can co-deliver drugs and PS while also transporting oxygen, thereby helping to alleviate tumor hypoxia-a major barrier to effective PDT [25,26].

To further improve therapeutic outcomes, recent research has focused on engineering liposomes with advanced functionalities. Two key strategies have emerged: (1) PS-drug co-delivery and [25,27], and (2) oxygen delivery to alleviate tumor hypoxia and boost ROS generation to achieve synergistic effects (Figure 1C) [28,29]. Other innovations include surface modifications for targeted delivery, integration with immunotherapy [30,31], and the development of theranostic platforms [32,33], all of which expand the therapeutic potential of liposome-based PDT.

This review begins by outlining the fundamental characteristics of liposomes, including their structural features, classifications, and historical development. Subsequently, the review discusses recent advancements in liposome engineering, including ligand-mediated targeting and the integration of multimodal therapeutic strategies. Next, it examines the mechanisms by which liposomes enhance PDT through PS and oxygen delivery and highlights the advantages of liposome-based PDT over conventional approaches. Finally, the review highlights the potential of functionalized liposomes as versatile theranostic platforms, with representative examples illustrating their applications in immunotherapy and combined diagnostic–therapeutic systems.

## 2. Liposome Structure and Preparation Method

Liposomes are artificially engineered lipid vesicles composed of a bilayer membrane surrounding an internal aqueous core [34], making them exceptional carriers for therapeutic agents. Their structural versatility and biocompatibility have prompted extensive research into optimizing their composition to overcome biological barriers and enhance targeted drug delivery [35]. Key strategies include controlling self-assembly, surface modification, incorporation of functional lipids, and maximizing drug loading efficiency [36,37].

**Figure 2:**
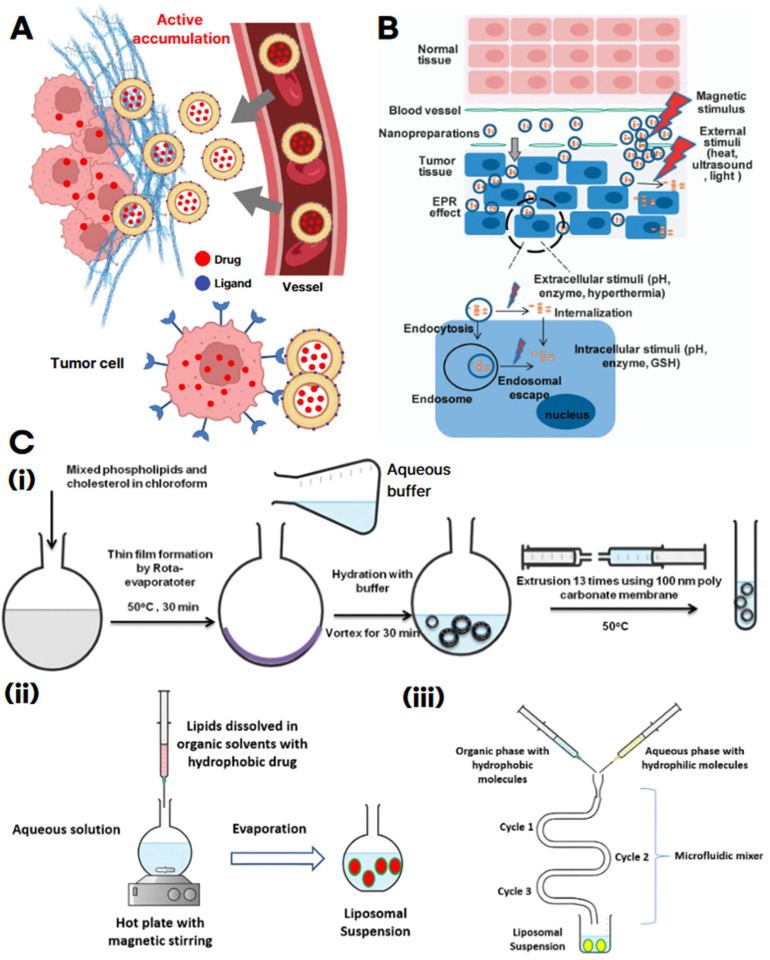
Liposome cancer targeting and preparation methods. (**A**) Schematic illustration of active targeting by ligand-modified liposomes, showing enhanced accumulation at the tumor site and selective drug delivery to tumor cells. The gray arrows indicate the preferential movement and accumulation of ligand-conjugated liposomes from blood vessels into tumor tissue, as well as their internalization into tumor cells. (**B**) Stimulus-responsive delivery strategy for tumor targeting. The red arrows represent external physical stimuli (e.g., magnetic field, ultrasound, heat, light) triggering drug release or cellular uptake. The gray arrows indicate the process of liposomal nanocarriers penetrating tumor tissue (EPR effect), entering tumor cells by endocytosis, escaping endosomes, and distributing drugs intracellularly in response to various stimuli. Reproduced with permission from [42]. (**C**) Schematic diagrams of major liposome preparation methods: (**i**) Thin-film hydration method, (**ii**) Solvent injection method, and (**iii**) Microfluidic channel. Black arrows sequentially indicate the process steps and directional flow of solutions and materials during liposome formation. Reproduced with permission from [53].

Tumor-targeting strategies represent a critical component in the development of liposomal formulations [38]. Among them, the most widely employed approach is active targeting, in which specific ligands (e.g., antibodies, peptides, small molecules, carbohydrates) are conjugated to the liposomal surface to selectively bind receptors overexpressed on cancer cells (Figure 2A) [38,39]. This strategy offers higher cellular specificity compared to passive targeting based solely on the enhanced permeability and retention (EPR) effect, and it facilitates efficient endocytosis into target cells [38,39,40]. Representative examples include folate receptor-, transferrin receptor-, and HER2-targeted liposomes, which have demonstrated improved tumor accumulation and therapeutic efficacy relative to non-targeted formulations. Nevertheless, key challenges remain, including potential immunogenicity of ligands, variability in receptor expression, and non-specific interactions with serum proteins, all of which can compromise reproducibility and clinical outcomes [41].

In contrast, stimulus-responsive targeting is designed to trigger drug release from liposomes in response to intrinsic tumor microenvironmental cues or external stimuli (Figure 2B) [42]. Tumors typically exhibit unique hallmarks such as hypoxia, acidic pH, elevated enzymatic activity (e.g., matrix metalloproteinases, phospholipases), and excessive levels of reactive oxygen species (ROS), which can be exploited as endogenous triggers for controlled release [42,43,44]. In addition, exogenous physical stimuli, including light, heat, ultrasound, and magnetic fields, have been utilized to induce on-demand drug release [44,45]. Such systems enhance the spatial and temporal precision of drug delivery, thereby minimizing damage to normal tissues and maximizing antitumor efficacy [45]. However, heterogeneity of tumor microenvironmental conditions, limited penetration depth of physical triggers, and safety concerns regarding repeated exposure remain significant barriers to translation [46].

Efficient and reproducible fabrication techniques are essential for clinical translation [46,47,48,49]. Among conventional methods, thin-film hydration remains the most widely used. In this approach, phospholipids and lipophilic drugs are dissolved in an organic solvent and subjected to rotary evaporation, forming a thin lipid film on the inner wall of a flask [46,50,51]. Hydration with an aqueous buffer at a temperature above the lipid phase transition point initiates self-assembly into multilamellar vesicles, encapsulating hydrophilic drugs within the aqueous core [51,52]. The resulting liposomes are then refined by extrusion or sonication to achieve the desired particle size and lamellarity (Figure 2C(i)).

Another commonly employed technique is the solvent injection method, wherein lipids dissolved in organic solvents are rapidly injected into an aqueous phase [54,55,56,57]. Solvent identity critically dictates lamellarity, size dispersion, and stability in solvent-injection products [55,58]. Although this method enables rapid production, it often results in a high polydispersity index (PDI) and potential particle instability due to residual solvents and elevated processing temperatures (Figure 2C(ⅱ)) [53].To address these limitations, microfluidic channel technology has emerged as a promising alternative [59,60,61]. This method utilizes precisely engineered microchannels to control the mixing of organic and aqueous phases at the microscale [62]. Phospholipids, typically dissolved in ethanol or isopropanol, are introduced into the microchannel where they encounter the aqueous stream. This controlled environment facilitates homogeneous mixing and leads to the formation of highly uniform liposomes [60,63,64]. The microfluidic approach offers superior control over critical parameters such as particle size, distribution, and lamellar structure, making it particularly suitable for producing monodisperse and reproducible liposomes for pharmaceutical applications (Figure 2C(ⅲ)) [53].

## 3. Liposome-Based PDT: PS and Drug Delivery Strategies

Liposomal platforms for PDT can be broadly categorized into four major application strategies: photosensitizer/drug co-delivery, hypoxia relief, immunotherapy integration, and theranostic design. Each category employs distinctive design principles to address the inherent limitations of PDT, including photosensitizer instability, tumor hypoxia, heterogeneous immune responses, and the need for image-guided treatment (Table 1). In the following sections, each category will be examined in detail, such as liposomal formulations for photosensitizers and drug delivery.

**Figure 3:**
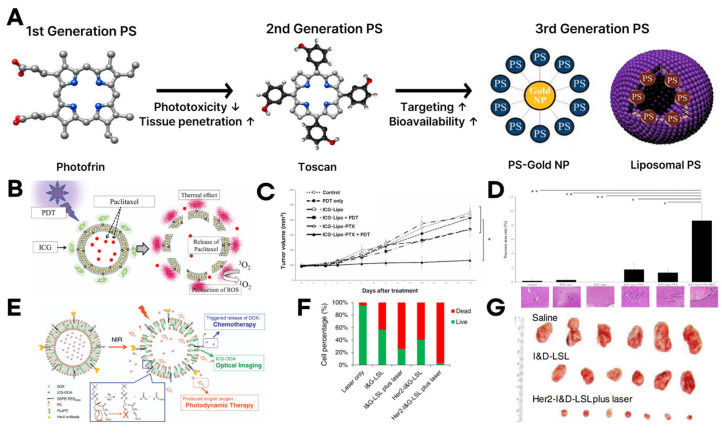
PS and drug delivery liposome-based PDT systems. (**A**) Schematic illustration of photosensitizer (PS) development, showing first-generation (Photofrin), second-generation (Toscan), and third-generation PS (PS-Gold NP, liposomal PS structure). Arrows indicate improvements in phototoxicity, tissue penetration, targeting, and bioavailability through each generation. Reproduced with permission from [65] (**B**) Schematic of the ICG-Lipo-PTX preparation. Green dots represent photosensitizer ICG, red dots represent drug paclitaxel. (**C**) Antitumor effects of ICG-Lipo in the subcutaneous tumor model inoculated with KPL-1 cells. (**D**) Pathological examination of subcutaneous tumors. Black bars indicate post-treatment tumor size, and purple indicates the extent of necrosis in histological images. Reproduced with permission from [75] (**E**) The illustration of the NIR light-mediated specific drug release and synchronous PDT and chemotherapy. Key functional regions are highlighted with matching colors to indicate chemotherapeutic agents (blue), optical imaging agents (green), and photosensitizers (orange). (**F**) Bar graph showing proportions of live (green) and dead (red) cells after treatments. (**G**) Photograph of SKOV3 tumors in each group at the end of the experiment. Reproduced with permission from [76].

The progression of photosensitizers provides important context for these liposomal strategies. These advances from first- to third-generation PSs have led directly to the improved delivery platforms, ultimately resulting in the integration of nanocarriers such as liposomes into PDT design [65]. PSs have undergone three generational advancements to address the limitations of earlier molecules [66,67,68]. First-generation PSs, such as Photofrin, suffered from poor tumor selectivity, limited absorption within the therapeutic window, and prolonged skin photosensitivity [19,67,69]. Second-generation PSs-including chlorin e6, hypericin, and phthalocyanines-overcame many of these drawbacks by operating in the 650–800 nm NIR range, enabling deeper tissue penetration [70,71,72]. These agents also exhibited higher singlet oxygen quantum yields, extended tumor retention, reduced side effects, and improved phototoxic specificity. However, their tumor selectivity remained suboptimal for fully optimized PDT. To enhance therapeutic precision, third-generation PSs were developed by conjugating PSs with targeting ligands (e.g., peptides or antibodies) or incorporating them into nanocarriers [68,73,74]. These strategies significantly improved tumor specificity, increased bioavailability, and minimized off-target toxicity (Figure 3A).

**Table 1:** Representative liposomal platforms designed for PDT.

Category	Main Function	System	Payload	Cell Type/Model	References
**PS/Drug delivery**	**Co-delivery (chemo–PDT synergy)**	ICG-Lipo-PTX	Indocyanine green (ICG) + Paclitaxel (PTX)	KPL-1 cell, BALB/c mouse	[75]
		DOX/ICG-Lipo	Indocyanine green (ICG) + Doxorubicin (DOX)	MCF-7/ADR breast cancer cell, mouse model	[76]
	**Phototriggered release (light/ROS-induced drug release)**	Porphyrin–phospholipid (POP)	PoP + small-molecule drug	Tumor xenograft; photo-triggered release under NIR	[77]
**Hypoxia relief**	**O_2_ delivery (external oxygen carriers)**	LIH-Lipo	Indocyanine green (ICG) + Hemoglobin (Hb)	4T1 breast cancer cell, mouse model	[78]
	**O_2_ generation (endogenous catalytic process)**	PPIX–MnO_2_ Lipo	Protoporphyrin IX (PPIX) + MnO_2_	MCF-7/HeLa, 4T1 tumor-bearing mice	[79]
		Catalase-Lipo	Catalase + Ce6	Hypoxic tumor model; catalytic O_2_ generation	[80]
**Immunotherapy**	**Chemo–PDT–induced ICD and immune activation**	GDPPL	Gemcitabine + DSPE-PEG-PheoA	Tumor-bearing mice	[81]
	**ICD amplification and immune activation (ER stress/antigen capture)**	PB Lipo (ER-biomimetic)	ER-biomimetic lipids + ICG	TNBC mouse model; PD-L1 blockade synergy	[82]
		IERL	Ce6 + catalase polymer with maleimide	Tumor-bearing mice; lung metastasis prevention	[83]
		Lipo-Ce6 (pyroptosis/ICD)	Ce6 (±adjuncts)	Solid tumor models; NLRP3/Caspase-1/activation	[84]
**Theranostic platforms**	**Hypoxia-activated chemo–PDT + miRNA imaging**	Lip/Ce6/TPZ-PmiRNA	Ce6 + tirapazamine (TPZ) + miRNA-155 probe	MCF-7 tumor-bearing mouse model	[85]
	**Dual-modal imaging-guided PDT (FLI/PAI)**	LBPD	PEGylated liposome + BPD	HeLa cells; tumor-bearing mice	[86]
	**Multimodal PDT + PTT + chemo, hypoxia/NTR responsive**	Gambogic acid (GA)/BN LIP	DSPE–AZO–PEG + GA + Bcy-NO_2_	Colorectal cancer mouse model	[87]
		HAP-theranostic Lipo	PS + hypoxia-activated prodrug (HAP)	Mouse tumor	[88]
		PMILs/BPD-Lipo (FL/PA guidance)	BPD (verteporfin) + multi-inhibitors	Murine xenografts	[89,90]

Among nanocarrier platforms, liposomes have emerged as particularly promising vehicles for PDT. Their biocompatible bilayer structure enables efficient encapsulation of both hydrophobic and hydrophilic PSs, enhancing solubility and stability while reducing aggregation [34,35,36]. Liposomes also facilitate selective delivery by minimizing nonspecific uptake, and their modular architecture supports co-delivery of PSs with chemotherapeutics or oxygen [18,35,91,92,93,94]. These features position liposomes as a central platform for next-generation PDT, capable of overcoming both delivery challenges and the multifactorial barriers of the tumor microenvironment.

A representative example involves the co-encapsulation of indocyanine green (ICG) and paclitaxel (PTX) within liposomes [95,96,97,98,99]. In this system, ICG is anchored to the liposomal membrane via a C18 chain, forming vesicles approximately 200 nm in diameter. Upon 810 nm NIR irradiation, ICG generates singlet oxygen and photothermal effects, destabilizing the liposomal bilayer and triggering localized PTX release (Figure 3B). This design enhances the photodynamic activity of ICG while ensuring efficient delivery of the chemotherapeutic agent [96]. Compared to controls, liposomal co-delivery of ICG and PTX combined with PDT resulted in pronounced tumor suppression (Figure 3C), with histological analysis revealing extensive tumor necrosis and confirming the superior therapeutic efficacy of the combined treatment system (Figure 3D) [76].

While previous systems relied on passive targeting, more advanced designs incorporate tumor-targeting antibody modifications to guide liposomes directly to cancer cells, thereby maximizing the synergistic effects of PDT and chemotherapy [66,90,98,99,100,101]. In one such design, a hydrophobic PS (ICG-ODA) was embedded in the liposomal membrane, doxorubicin (DOX) was encapsulated in the aqueous core, and the surface was functionalized with anti-HER2 antibodies for tumor-specific targeting (Figure 3E) [100,102]. In vitro studies demonstrated that HER2-targeted liposomes induced extensive cancer cell death under NIR irradiation (Figure 3F), while in vivo experiments showed marked tumor suppression in mouse models (Figure 3G) [76].

Both studies share the common strategy of utilizing liposomes for PDT with chemotherapy. These studies have shortcomings; ICG-Lipo-PTX has the limitation of insufficient targeting specificity, whereas DOX/ICG-Lipo demonstrates superior tumor selectivity and precise control but still requires improvements in safety and large-scale manufacturing for clinical translation [75,76]. Despite their disadvantages and distinctive designs of liposomes, they show the therapeutic potential of liposome-based PDT–chemotherapy combination therapy, despite employing distinct design strategies.

Currently, various liposomal formulations of chemotherapeutic agents are already in clinical use, with Doxil^®^ (liposomal doxorubicin) and Onivyde^®^ (liposomal irinotecan) being representative examples [103,104]. In addition, indocyanine green (ICG) has been applied in surgical imaging and evaluated in several clinical trials for PDT, while near-infrared photoimmunotherapy (NIR-PIT) using an EGFR-targeted antibody–IR700 conjugate was approved [105]. Nevertheless, ICG-liposome-based combination systems remain at the preclinical stage, with challenges such as uncertain in vivo distribution and metabolism, the lack of standardized irradiation protocols and equipment, and the need for comprehensive long-term toxicity evaluations [105,106]. For successful clinical translation, it will be essential to develop tumor-specific targeting strategies and to optimize irradiation conditions suitable for clinical practice [106,107,108,109]. Encouragingly, several research efforts are already progressing toward early-phase clinical trials.

## 4. Liposome-Based PDT: Hypoxia-Relief Strategies

The tumor microenvironment (TME) plays a critical role in tumor progression, therapeutic resistance, and malignancy [110]. Characterized by hypoxia, oxidative stress, and acidosis, the TME alters the extracellular matrix (ECM) and disrupts angiogenic and immune responses, creating a niche that supports tumor growth and survival [111]. These pathological features pose significant challenges to the efficacy of various cancer therapies, including PDT [110,112,113,114]. In hypoxic tumors, limited oxygen availability severely impairs ROS generation, thereby diminishing the therapeutic impact of PDT [115,116]. Moreover, PDT itself can exacerbate hypoxia by consuming local oxygen and inducing vascular damage. This secondary hypoxia may further compromise therapeutic efficacy and promote tumor progression and metastasis [110,117,118,119,120]. Therefore, strategies to alleviate hypoxia are essential for improving PDT outcomes.

**Figure 4:**
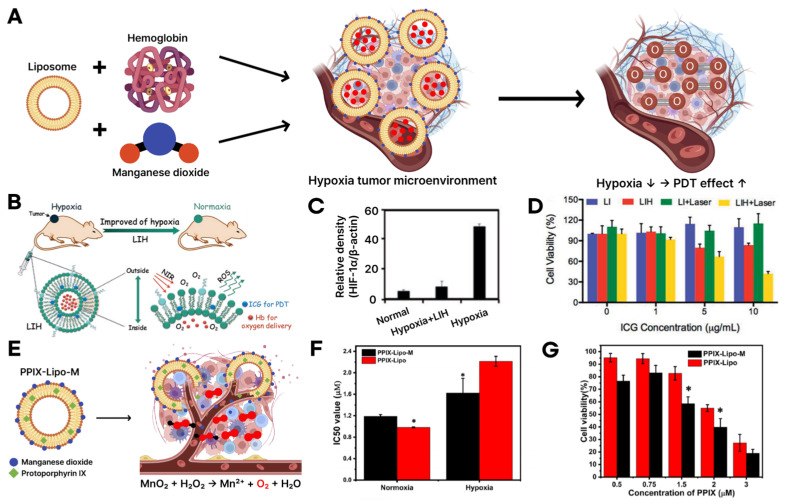
(**A**) Schematic illustration of design for hemoglobin or manganese dioxide-loaded liposomes for overcoming tumor hypoxia. The arrows in this panel indicate the direction of process flow from individual components to the improved tumor microenvironment and subsequent enhancement of PDT effects. (**B**) Schematic illustration of reduction in tumor hypoxia and enhanced PDT based on photosensitizer and hemoglobin co-loaded liposomes (LIH). Green circle indicates liposome that induces hemoglobin, and green arrows show the process of alleviating tumor hypoxia and promoting the generation of a large amount of reactive oxygen species (ROS). (**C**) Semi-quantitative analysis of HIF-1a expression in CT-26 cell lines. (**D**) Cytotoxicity of LI and LIH against CT-26 cells in hypoxia environment without or with laser irradiation (808 nm, 1 W/cm^2^, and 1 min). Blue bar indicates group LI, red bar indicates group LIH, green bar indicates group LI+Laser, and yellow bar indicates group LIH+Laser. Reproduced with permission from [78] (**E**) Schematic illustration of PPIX and manganese dioxide co-loaded liposomes mediating in situ oxygen generation in the TME. Yellow circles represent liposomes, blue circles represent manganese dioxide, green square represent protoporphyrin IX, and red circles represent oxygen. The arrows depict the transformation of manganese dioxide (MnO_2_) with hydrogen peroxide (H_2_O_2_) and oxygen (O_2_) generation, leading to improved therapeutic efficacy. (**F**) IC-50 values of PPIX-Lipo and PPIX-Lipo-M to MCF-7 cell lines and incubated for 24 h. (**G**) PDT effect of PPIX-Lipo and PPIX-Lipo-M under hypoxia. Reproduced with permission from [79].

To address these limitations, researchers have developed liposomal nanoplatforms that integrate oxygen-delivering hemoglobin or oxygen-generating manganese dioxide (MnO_2_) with PSs [121,122,123,124]. These systems are designed to either deliver oxygen directly to hypoxic tumor sites or catalytically generate oxygen in situ, thereby modulating the TME and enhancing PDT efficacy (Figure 4A) [125]. One representative approach involves the co-delivery of ICG and hemoglobin in a liposomal formulation (LIH). This system supplies oxygen to tumor tissues and activates ICG under NIR irradiation to produce ROS, thereby amplifying oxidative damage to cancer cells (Figure 4B). In vitro analyses demonstrated that LIH treatment effectively reduced hypoxia, as evidenced by downregulation of hypoxia-inducible factor 1-alpha (HIF-1α) expression (Figure 4C). Furthermore, the system exhibited strong synergistic cytotoxicity under laser irradiation, confirming its therapeutic potential against hypoxic tumors (Figure 4D) [78].

In a complementary strategy, liposomes incorporating protoporphyrin IX (PPIX) as a PS were engineered with MnO_2_ on their surface to catalyze oxygen generation [111,126,127]. Upon encountering tumor-localized hydrogen peroxide, MnO_2_ decomposes it into molecular oxygen, thereby elevating local oxygen levels and sustaining ROS production (Figure 4E). Under hypoxic conditions, PPIX–MnO_2_ liposomes exhibited significantly lower IC50 values than control formulations, indicating superior cytotoxicity driven by enhanced oxygen availability (Figure 4F). Additionally, cell viability assays demonstrated greater therapeutic efficacy of PPIX–MnO_2_ liposomes, particularly at higher PPIX concentrations, validating their potential for robust ROS generation and improved PDT performance (Figure 4G) [79].

Hemoglobin-based LIH liposomes and MnO_2_ liposomes, which generate oxygen internally, differ in their strategies for alleviating tumor hypoxia. LIH liposomes deliver oxygen directly from an external source, thereby inducing rapid and potent reactive oxygen species (ROS) generation; however, they require repeated administration and exhibit limitations related to in vivo stability. In contrast, MnO_2_ liposomes continuously produce oxygen by utilizing the biochemical reactions within the tumor microenvironment, yet they face challenges such as potential metal ion accumulation and long-term toxicity. Recently, hybrid liposomal systems that integrate the advantages of both approaches have been developed, enabling the simultaneous functions of external oxygen delivery and internal oxygen generation [78,79]. Future studies should focus on quantitatively evaluating oxygen delivery efficiency and biosafety. Taken together, these contrasting strategies underscore a fundamental design trade-off: hemoglobin-based liposomes provide rapid yet transient oxygen delivery that enhances immediate PDT efficacy, whereas MnO_2_-modified systems offer sustained oxygen generation but raise concerns regarding long-term stability and potential toxicity. Hybrid designs that integrate both logics are promising, yet their scalability and biosafety require rigorous validation. Ultimately, the translational success of hypoxia-relieving liposomal PDT platforms will depend on balancing immediacy, durability, and safety within clinically feasible manufacturing frameworks, as well as establishing standardized manufacturing processes to enhance the translational potential of hypoxia-alleviating PDT platforms [128,129,130].

## 5. Liposome-Based PDT: Integration with Immunotherapy

While early efforts in liposomal PDT primarily focused on enhancing PS delivery, improving tumor accumulation, and mitigating hypoxia-related limitations [19,131,132,133], recent advances have expanded the scope to include immunotherapeutic strategies [134,135,136]. Beyond their established role in ROS-mediated tumor ablation, contemporary liposomal platforms are now engineered to function as immunodulatory agents-promoting immunogenic cell death (ICD), activating antigen-presenting cells (APCs), and triggering systemic antitumor responses [137,138,139]. These approaches seek to harness PDT-induced immune mechanisms in a controlled and targeted manner, transitioning from localized tumor destruction to systemic immune activation [140,141]. Recent studies have introduced diverse liposomal designs that incorporate chemotherapeutic agents [142,143], immune checkpoint inhibitors [144], and vaccine adjuvants to amplify antitumor immunity [145].

**Figure 5:**
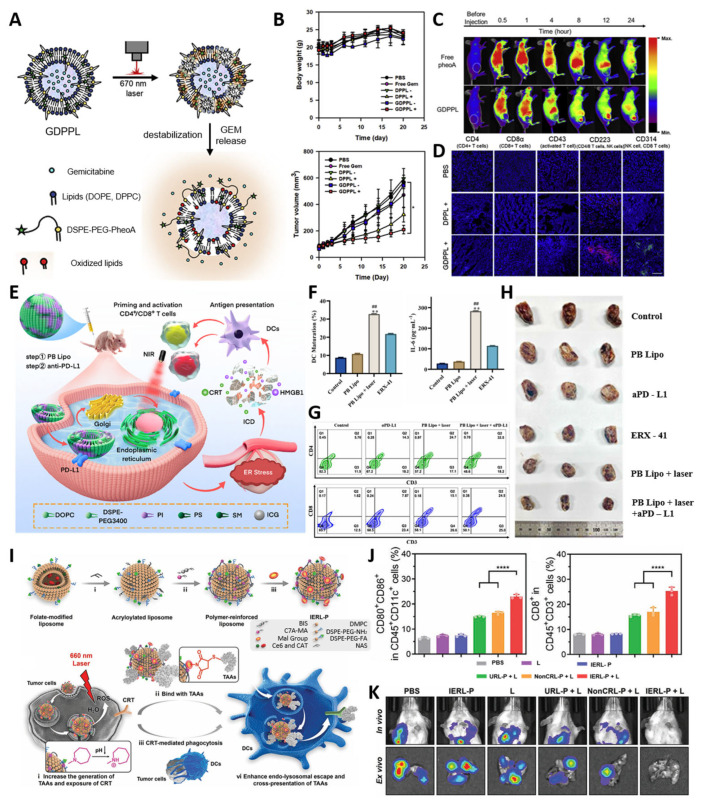
Liposome-based PDT–immunotherapy systems. (**A**) Schematic of gemcitabine-loaded DSPE-PEG-PheoA liposomes (GDPPLs) and their light-triggered drug release mechanism. (**B**) Tumor volume and body weight changes in HuCCT-1 tumor-bearing mice following various treatments (* *p* < 0.001), tumor volume was remarkably reduced in the GDPPL+ groups, demonstrating effective tumor suppression, while body weight remained stable across all groups, indicating minimal systemic toxicity.(**C**) Flow cytometry analysis of immune cell populations (CD4^+^ T cells, CD8^+^ T cells, and NK cells) in tumor post-treatment. (**D**) Enhanced infiltration of CD4^+^ and CD8^+^ T cells in tumor tissues of BALB/c mice following PB Lipo + laser + αPD-L1 therapy. Reproduced with permission from [81] (**E**) Schematic of ER-targeted PB Lipo with ICG for ROS-mediated immunogenic cell death (ICD) and immune activation with PD-L1 blockade. (**F**) Enhanced dendritic cell maturation and elevated IL-6 secretion following PB Lipo + laser treatment, compared with both the no-laser (** *p* < 0.01) and positive-control groups (## *p* < 0.01). (**G**) Increased CD4^+^ and CD8^+^ T cell infiltration in tumors following PB Lipo + laser + αPD-L1 therapy. (**H**) Significant tumor growth inhibition in TNBC models, with the most pronounced effect observed in the PB Lipo + laser + αPD-L1 group. Reproduced with permission from [82] (**I**) Schematic of polymer-reinforced liposomes (IERLs) and their mechanism of action. (**J**) Increased dendritic cell maturation and CD8^+^ T cell infiltration in tumors after IERL-based PDT, compared with both the no-laser and positive-control groups (**** *p* < 0.0001). (**K**) Bioluminescence imaging showing reduced tumor recurrence and lung metastasis. Reproduced with permission from [83].

Kim et al. [81] developed a gemcitabine-loaded DSPE-PEG-PheoA liposome (GDPPL) that co-delivers PDT agents and chemotherapeutic agents to achieve simultaneous cytotoxicity and immune activation [144,145]. Upon light irradiation, ROS-mediated lipid peroxidation destabilizes the bilayer, accelerating gemcitabine release and amplifying PDT-induced tumor cell death (Figure 5A). This formulation resulted in pronounced tumor regression and robust infiltration of CD4^+^/CD8^+^ T cells and natural killer (NK) cells, outperforming both free drug and PDT monotherapy (Figure 5B,C,D) [146,147,148]. By stabilizing gemcitabine, ensuring spatiotemporal release, and provoking immunogenic cell death, GDPPL functioned not only as a combined chemo–PDT platform but also as an immune adjuvant, underscoring its potential to overcome drug inactivation, enhance antitumor immunity, and address the immunosuppressive tumor microenvironment [137,140].

Li et al. [82] introduced endoplasmic reticulum (ER)-biomimetic liposomes (PB Lipo) encapsulating ICG for organelle-specific targeting and enhanced PDT efficacy (Figure 5E) [149]. By mirroring key ER phospholipids, PB Lipo preferentially localizes to the ER, and upon NIR light activation, provokes robust ER stress that drives ROS-mediated damage and immunogenic cell death (ICD), evidenced by calreticulin exposure and HMGB1/ATP release with dendritic-cell maturation and pro-inflammatory cytokine production (Figure 5F) [134,138]. In triple-negative breast cancer models, combining PB Lipo–PDT with PD-L1 blockade markedly increased intratumoral CD4^+^/CD8^+^ T-cell infiltration and achieved ~79% tumor growth inhibition (Figure 5G,H) [141]. Together, these data indicate that ER-directed liposomal PDT can precisely position the photosensitizer at an immunogenic organelle to amplify antigen presentation and synergize with immune checkpoint blockade for potent cancer immunotherapy.

Zhao et al. [83] developed a polymer-reinforced liposome (IERL) designed to enhance PDT-induced ICD and subsequent antitumor immunity (Figure 5I) [135,139].

By integrating a thin crosslinked polymer network onto a folate-targeted liposomal bilayer and incorporating maleimide groups for antigen capture, IERLs provide a bioactive interface that covalently captures as-generated tumor-associated antigens (TAAs) and, via proton-sponge C7A moieties, facilitate endo-lysosomal escape to boost cross-presentation in dendritic cells [145], generate robust ROS and oxygen, driving ICD characterized by calreticulin exposure/HMGB1 release, enhanced antigen cross-presentation, and DC maturation (CD80/CD86 upregulation), with substantial intratumoral CD8^+^ T-cell infiltration (Figure 5J) [146,147]. This strategy suppressed primary tumor growth, elicited systemic immunity with an abscopal effect, and inhibited lung metastasis upon tumor rechallenge-indicating durable immune memory (Figure 5K) [148]; moreover, outcomes were further improved when combined with anti-PD-1 therapy. Collectively, IERL exemplifies how polymer-reinforced, antigen-capturing liposomes can extend conventional PDT beyond local cytotoxicity to durable, system-wide antitumor immunity [150].

Recent advances in liposome-based PDT demonstrate how rational design can synergize with immunotherapy to achieve both local tumor control and systemic immune activation. Across the three representative systems, all platforms converged on inducing immunogenic cell death (ICD) and promoting CD4^+^/CD8^+^ T-cell infiltration, yet each pursued distinct design logics: gemcitabine–PheoA liposomes prioritized drug stability and dual chemo–PDT action, ER-biomimetic liposomes leveraged organelle selectivity to amplify ER stress-driven ICD and checkpoint blockade synergy, and polymer-reinforced antigen-capturing liposomes established durable immune memory by stabilizing vesicle architecture and retaining tumor-associated antigens. These complementary strategies collectively highlight the versatility of liposomal engineering in overcoming the transient and localized nature of conventional PDT. Nonetheless, significant challenges remain for clinical translation, including variability of immune responses across tumor types, the need for reproducible large-scale manufacturing, and rigorous evaluation of long-term biosafety, particularly regarding immune overactivation and chronic toxicity. To bridge these gaps, future work should focus on integrating theranostic functionalities for real-time monitoring, developing standardized irradiation and dosing protocols, and tailoring liposomal architectures through biomarker-driven personalization. Such efforts will be crucial for establishing liposomal PDT–immunotherapy platforms as clinically viable and patient-specific cancer treatments.

## 6. Liposome-Based PDT: Theranostics

Theranostic platforms, which integrate therapeutic functions with real-time imaging, offer significant advantages for PDT [150,151]. They enable precise tumor localization, monitoring of PS distribution, and dynamic evaluation of therapeutic response-capabilities that are particularly valuable given the inherent unpredictability of PDT outcomes due to limited light penetration, uneven PS biodistribution, and tumor hypoxia [152]. By embedding these functions into liposomal nanocarriers, diverse payloads and imaging probes can be co-delivered in a structurally tunable format, allowing integration of multiple diagnostic and therapeutic cues within a single vesicle. Through multimodal imaging capabilities-such as fluorescence, photoacoustic, and magnetic resonance imaging-theranostic liposomes enable personalized treatment planning and facilitate image-guided irradiation protocols [153,154]. These features are expected to facilitate closed-loop cancer therapy, where diagnosis and treatment are seamlessly integrated [155].

**Figure 6:**
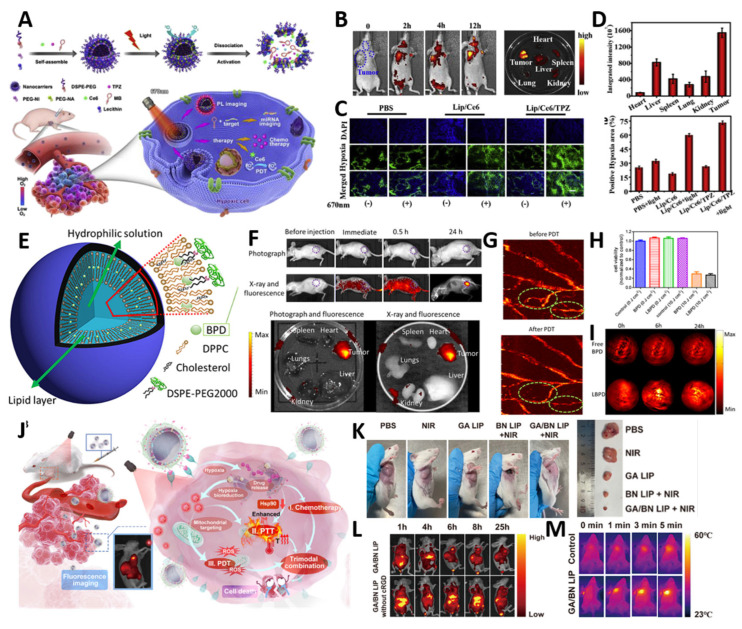
Multifunctional liposomes for theranostic applications. (**A**) Schematic of hypoxia-responsive liposomes (Lip/Ce6/TPZ–PmiRNA) for simultaneous tumor diagnosis and synergistic PDT–chemotherapy. (**B**) In vivo fluorescence images showing time-dependent tumor accumulation of Lip/Ce6/TPZ nanoparticles post-intravenous injection. (**C**) Ex vivo fluorescence biodistribution of major organs and tumors at 12 h post-injection, confirming tumor-specific accumulation. (**D**) Immunofluorescence staining of tumor sections showing enhanced hypoxia post-PDT, validating TPZ activation. Reproduced with permission from [85] (**E**) Schematic of PEGylated liposomal benzoporphyrin derivative monoacid ring-A (LBPD), showing its structural components and theranostic function. (**F**) In vivo and ex vivo fluorescence images demonstrating tumor-specific LBPD accumulation at 24 h post-injection. Purple circles denote the tumor tissue. (**G**) Photoacoustic vascular imaging before and after LBPD-mediated PDT, showing vessel damage and structural disruption. The green circles denote the significant changes to the vascular structures. (**H**) In vitro PDT efficacy of LBPD in HeLa cells, with significantly reduced cell viability under 690 nm laser irradiation. (**I**) In vivo photoacoustic imaging of tumor sites post-LBPD administration, showing time-dependent accumulation at 0, 6, and 24 h. Reproduced with permission from [86] (**J**) Schematic of the multifunctional liposomal system, showing the encapsulated agents and their synergistic roles in photodynamic and photothermal therapy. (**K**) Representative in vivo and ex vivo photographs of tumor-bearing mice after different treatments. Visible tumor shrinkage in mice and markedly reduced excised tumor sizes were observed post-treatment. (**L**) Fluorescence and photoacoustic imaging results confirming liposome accumulation and enabling theranostic monitoring. The red circle denote the tumor location. (**M**) Photothermal imaging following laser irradiation, demonstrating efficient localized heat generation for tumor ablation. Reproduced with permission from [87].

Zhang et al. [85] developed a hypoxia-responsive theranostic liposome (Lip/Ce6/TPZ-PmiRNA) that co-delivers Ce6, the hypoxia-activated prodrug tirapazamine (TPZ), and an miRNA-155 molecular beacon probe for tumor-specific diagnosis (Figure 6A) [150,155]. Upon Ce6-mediated PDT, intratumoral oxygen is consumed, creating a hypoxic microenvironment that triggers degradation of the PEG–2–nitroimidazole (PEG–NI)-based hypoxia–sensitive polymer in the liposomal shell, thereby activating TPZ [156,157,158]. Simultaneously, the released miRNA–155 probe hybridizes with its target sequence in tumor cells, emitting a fluorescence signal that enables tumor detection [159]. In an MCF–7 tumor–bearing mouse model, fluorescence imaging revealed a progressive increase in signal intensity at the tumor site, peaking at 12 h post–injection (Figure 6B), which was further confirmed by ex vivo biodistribution analysis showing preferential tumor accumulation (Figure 6C) [160]. Immunofluorescence staining demonstrated a significantly higher proportion of hypoxic regions in PDT–treated tumors compared to non-irradiated controls (Figure 6D), providing favorable conditions for TPZ activation. Collectively, this system achieved potent synergistic effects by combining PDT with hypoxia-activated chemotherapy while simultaneously providing molecular-level diagnostic readouts, illustrating the potential of theranostic liposomes to overcome the oxygen-dependence of PDT and advance toward clinically relevant image-guided interventions [155].

Xu et al. [86] designed a theranostic nanoplatform encapsulating the PS benzoporphyrin derivative monoacid ring-A (BPD) within PEGylated nanoliposomes (LBPD) to enable dual-modal fluorescence (FLI) and photoacoustic imaging (PAI)-guided PDT (Figure 6E). Encapsulation in PEGylated liposomes enhanced tumor accumulation through prolonged circulation and EPR-driven uptake, minimizing off-target distribution, as confirmed by strong FLI signals at tumor sites (Figure 6F) and pronounced PAI enhancement up to 24 h post-injection (Figure 6I) [153,154]. Guided by imaging, LBPD-mediated PDT effectively disrupted tumor vasculature, and prognosis could be monitored in real time via photoacoustic mapping of vascular changes (Figure 6G) [161]. In vitro assays confirmed potent cytotoxicity against HeLa cells, with cell viability reduced to ~27% (Figure 6H), comparable to free BPD. This study highlights how clinically approved BPD, when reformulated into PEGylated liposomes, acquires dual diagnostic and therapeutic functions, enabling image-guided vascular-targeted PDT with improved safety and translational feasibility.

Wu et al. [87] developed a hypoxia–responsive theranostic liposomal platform (GA/BN LIP) that integrates chemotherapy, photothermal therapy (PTT), and PDT with real–time fluorescence imaging for colorectal cancer treatment [162,163]. The azo–linked amphiphilic lipid (DSPE–AZO–PEG) formed a hypoxia–cleavable shell, further modified with cRGD peptides for αvβ3–mediated tumor targeting [164,165]. The liposome co–encapsulated gambogic acid (GA), a natural HSP90 inhibitor that suppresses heat–shock-mediated thermotolerance, and Bcy–NO_2_, a nitroreductase (NTR)-responsive heptamethine cyanine dye capable of mitochondrial monitoring. Under hypoxic conditions, azoreductase cleavage triggered rapid payload release, while NTR–catalyzed reduction restored the fluorescence of Bcy–NO_2_, enabling real-time imaging and mitochondrial localization [166,167,168]. In vivo studies demonstrated that GA/BN LIP combined with NIR irradiation led to significant tumor regression (Figure 6K), accompanied by strong tumor–localized fluorescence and photothermal signals (Figure 6L). A temperature increase of ~55 °C (Figure 6M) confirmed efficient photothermal conversion. This trimodal strategy overcame both hypoxia-related PDT inefficiency and PTT resistance, delivering potent antitumor effects with concurrent imaging capability, thereby underscoring the promise of multifunctional liposomal designs for precise, image-guided therapy [169].

Theranostic liposomal PDT platforms present a compelling strategy for integrating targeted therapy with real-time diagnostic monitoring, enabling image-guided treatment and dynamic assessment of therapeutic outcomes [150,155]. Hypoxia-responsive designs (Ce6/TPZ with a PEG–NI shell and a miRNA-155 probe) synchronize therapy with on-treatment physiology, dual-modal FL/PA liposomes (LBPD) couple vascular-level mapping to light delivery, and trimodal GA/BN LIP integrates chemotherapy–PTT–PDT under hypoxia-cleavable control. These studies imply that imaging readouts can gate dosing and timing for adjuvant therapies. These diverse approaches leverage the structural versatility of liposomes to combine multiple imaging modalities with therapeutic payloads, allowing adaptation to the heterogeneous tumor microenvironment [153,154]. However, despite their promise, clinical translation faces several challenges, including complex fabrication processes, scalability limitations, and stringent regulatory requirements associated with multi-component nanomedicines. Priority technical needs include batch-to-batch reproducibility of multi-payload assemblies, quality control of trigger/probe activation thresholds, and harmonization of imaging–irradiation parameters for protocol standardization; clinically, heterogeneity of immune and hypoxia responses mandates on-treatment imaging–guided adaptation rather than one-size-fits-all dosing. Addressing these hurdles will require the development of simplified and standardized manufacturing protocols, robust preclinical models for long-term evaluation, and clear regulatory frameworks that accommodate the dual diagnostic–therapeutic nature of these systems [170,171] so that theranostic liposomes can progress from proof-of-concept toward reproducible, image-guided care pathways.

## 7. Conclusions

In this review, we summarized the fundamental principles of PDT and examined the evolution of liposomal platforms beyond their conventional role as passive drug carriers. Our discussion encompassed liposomal strategies for precise PS/drug delivery, engineering approaches to alleviate tumor hypoxia, immunotherapy-integrated liposomes designed to induce ICD and enhance antitumor responses, and theranostic platforms that combine diagnostic imaging with therapeutic functions. Collectively, these innovations position liposome-assisted PDT as both a potent therapeutic modality and a cornerstone of precision oncology.

Despite these advances, translation into clinical practice remains hindered by several persistent challenges. The large-scale, reproducible manufacturing of multi-component liposomes is technically demanding, and maintaining stability during long-term storage continues to be unresolved. Comprehensive biosafety and pharmacokinetic evaluations, particularly regarding immune heterogeneity and the risks of chronic toxicity, should be conducted for translational success. Moreover, regulatory frameworks are not yet fully equipped to evaluate nanomedicines that combine drugs, photosensitizers, and diagnostic probes within a single construct, creating uncertainty in approval pathways.

Looking ahead, future efforts must simplify liposomal architectures without compromising multifunctionality, establish standardized GMP-compatible protocols, and develop robust preclinical models that capture long-term efficacy and biosafety. In addition, overcoming current targeting limitations–including ligand immunogenicity, receptor heterogeneity, and insufficient control of stimulus penetration–will be pivotal for clinical translation. Future efforts may combine ligand-guided specificity with microenvironment-responsive release mechanisms to achieve dynamic and patient-tailored tumor targeting.

One of the potential approaches toward clinical translation is closed-loop system, which integrates theranostic and real-time imaging feedback, Biomarker-guided personalization and AI-assisted image analysis may enable adaptive protocols tailored to tumor heterogeneity, contributing to the clinical practice of liposome-based therapy If these challenges are systematically addressed, liposomal PDT systems may progress from preclinical innovation to clinical reality, uniting therapeutic efficacy, immune modulation, and diagnostic precision as integral components of personalized oncology.
